# Oxidised Albumin Levels in Plasma and Skeletal Muscle as Biomarkers of Disease Progression and Treatment Efficacy in Dystrophic *mdx* Mice

**DOI:** 10.3390/antiox13060720

**Published:** 2024-06-13

**Authors:** Jessica R. Terrill, Angelo Patrick R. Bautista, Irene Tsioutsias, Miranda D. Grounds, Peter G. Arthur

**Affiliations:** 1 School of Molecular Sciences, The University of Western Australia, Perth, WA 6009, Australia; jessica.terrill@uwa.edu.au (J.R.T.); angelo.bautista@research.uwa.edu.au (A.P.R.B.); irene.tsioutsias@uwa.edu.au (I.T.); 2 School of Human Sciences, The University of Western Australia, Perth, WA 6009, Australia; miranda.grounds@uwa.edu.au

**Keywords:** Duchenne muscular dystrophy, *mdx* mice, redox modifications, albumin, albumin oxidation, biomarkers, taurine

## Abstract

Redox modifications to the plasma protein albumin have the potential to be used as biomarkers of disease progression and treatment efficacy in pathologies associated with inflammation and oxidative stress. One such pathology is Duchenne muscular dystrophy (DMD), a fatal childhood disease characterised by severe muscle wasting. We have previously shown in the *mdx* mouse model of DMD that plasma albumin thiol oxidation is increased; therefore, the first aim of this paper was to establish that albumin thiol oxidation in plasma reflects levels within *mdx* muscle tissue. We therefore developed a method to measure tissue albumin thiol oxidation. We show that albumin thiol oxidation was increased in both *mdx* muscle and plasma, with levels correlated with measures of dystropathology. In dystrophic muscle, albumin content was associated with areas of myonecrosis. The second aim was to test the ability of plasma thiol oxidation to track acute changes in dystropathology: we therefore subjected *mdx* mice to a single treadmill exercise session (known to increase myonecrosis) and took serial blood samples. This acute exercise caused a transient increase in total plasma albumin oxidation and measures of dystropathology. Together, these data support the use of plasma albumin thiol oxidation as a biomarker to track active myonecrosis in DMD.

## 1. Introduction

Albumin is the most abundant plasma protein and is present in almost all mammalian tissues, with approximately 60% of total body albumin in the extravascular compartment of muscle, skin, and adipose tissue [1,2]. Apart from its roles in osmosis, signalling, and transport, albumin is considered an important antioxidant due to the free thiol group of cysteine 34 (Cys34), allowing this to serve as a trap for reactive oxygen and nitrogen species [3]. Cys34 exists mostly in a reduced state in human plasma but is susceptible to direct oxidation by oxidants or indirect oxidation via thiol/disulfide (SH/SS) exchange reactions [4,5,6]. Cys34 has three isoforms according to the redox state of the free cysteine residue at position 34: mercaptalbumin (reduced albumin), non-mercaptalbumin-1 (reversibly oxidised albumin), and non-mercaptalbumin-2 (irreversibly oxidised albumin) [7]. It should be noted that in mice only, we have observed an additional cysteine residue on albumin that is susceptible to redox modifications; whereas in humans, rats, dogs, cows, horses, and sheep, we have observed only one (which is consistent with previous research). Oxidative modifications of serum albumin Cys34 have previously been investigated in exercise [8] and various disease states, such as organ failure, kidney diseases, and diabetes mellitus [4,9,10,11,12,13,14], where increased percentages of reversibly and irreversibly oxidised plasma albumin Cys34 are reported.

We have investigated the use of plasma albumin thiol oxidation as a blood biomarker in animal models of Duchenne muscular dystrophy (DMD), a fatal X-chromosome linked disease with an incidence of 1 in 5000 male births (reviewed in [15,16,17]). DMD occurs due to mutations in the dystrophin gene that result in dysfunctional or missing dystrophin protein in skeletal muscle [18]. An absence of functional dystrophin leads to a severe loss of muscle mass over time, due to increased susceptibility of myofibres to sarcolemma damage resulting in myofibre necrosis (myonecrosis) (reviewed in [19,20]). While the precise mechanisms of myonecrosis and progressive dystropathology remain unclear, oxidative stress caused by excessive generation of oxidants has long been widely implicated (reviewed in [21,22,23,24,25]). We recently investigated the relationship between myonecrosis and oxidative stress in dystrophic muscle and showed images of protein oxidation mainly colocalised to areas of myonecrosis and associated immune cell infiltration [26]. We also showed that in the *mdx* mouse model for DMD [27], plasma albumin thiol oxidation is increased at various stages of the disease and correlates with plasma creatine kinase (CK) levels, a marker of dystropathology [28].

Our research into this potential blood biomarker for DMD hypothesised that the increased albumin thiol oxidation in *mdx* plasma is due to increased oxidative stress associated with myonecrosis within the large mass of dystrophic muscle. The primary aim of the present study was to test this hypothesis, by examining the relationship between plasma and muscle albumin thiol oxidation using *mdx* mice. To facilitate this, we modified the plasma thiol oxidation technique developed in our laboratory [28] to be suitable for tissue analysis of albumin oxidation by immunoblot. We then measured albumin thiol oxidation in plasma and muscle from *mdx* and normal control C57 (WT) mice at two ages: 23 days (when myonecrosis as a measure of dystropathology is most severe) and 12 weeks (when myonecrosis is less active). We also included a group of 23-day-old *mdx* mice treated with taurine, since we and others have shown that taurine is an effective therapeutic intervention for this muscular dystrophy, including reduced myonecrosis [29,30,31,32,33,34,35,36,37].

We compared the levels of albumin thiol oxidation with albumin protein content and measures of dystropathology such as myofibre necrosis, muscle inflammation (myeloperoxidase activity), and membrane leakiness, as measured by plasma CK [38]. An additional aim was to further test the use of plasma albumin thiol oxidation as a biomarker of myonecrosis and dystropathology, in response to an acute intervention known to modify the amount of myonecrosis. Since we could not take serial blood samples from 23-day-old young mice subjected to taurine treatment (such juvenile mice are too small), we measured albumin thiol oxidation in plasma taken from tail-vein samples from 12 week adult *mdx* mice subjected to a single treadmill exercise session, known to increase dystropathology [32,39]. We also tracked plasma CK and grip strength in these mice, as established biomarkers of muscle pathology.

This research developed a simple new method to measure albumin thiol oxidation in tissues. This was used as a marker of oxidative stress in dystrophic muscle, to provide more evidence of a strong relationship between plasma and muscle albumin thiol oxidation (and other measures of dystropathology). This study confirms that plasma albumin thiol oxidation is a reliable biomarker that can be used to track levels of myonecrosis in *mdx* mice.

## 2. Materials and Methods

All chemicals and reagents were purchased from Sigma-Aldrich, St Louis, MO, USA, unless otherwise stated.

### 2.1. Animal Procedures

Experiments were carried out on dystrophic *mdx* (C57Bl/10ScSn*^mdx/mdx^*) and normal wildtype control (C57Bl/10ScSn) mice (the parental strain for *mdx*) from the Animal Resource Centre, Murdoch, Western Australia. Mice were maintained at the University of Western Australia under standard conditions, with free access to food and drinking water. All experiments were conducted in strict accordance with the guidelines of the National Health and Medical Research Council Code of practice for the care and use of animals for scientific purposes (2004) and the Animal Welfare act of Western Australia (2002) and were approved by the Animal Ethics committee at the University of Western Australia (ethics number 2020ET000034). Young mice were sampled at 23 days of age and adult mice at 12 weeks.

### 2.2. Taurine Treatment

Juvenile *mdx* mice were given taurine from 15 days of postnatal age (prior to weaning and the acute onset of myonecrosis that occurs by 21 days) in soft chow containing 4% taurine. Untreated *mdx* and WT mice had soft chow without taurine. Each group included pups (*n* = 8), with approximately equal male and female *mdx* pups, with all males for the WT group. Mice were sampled at 23 days of age after 7 days of taurine treatment.

### 2.3. Treadmill Exercise

Adult *mdx* mice aged 12 weeks (n = 7–8) underwent a single 30 min exercise session on a horizontal rodent treadmill (Columbus Instruments, Columbus, OH, USA), using an established protocol [39]. In brief, the protocol involved a settling (stationary) period for 2 min, an acclimatisation with gentle walking period for 2 min (2 m/minute), a warm-up period for 8 min (8 m/minute) and the main exercise session of 30 min at a pace of 12 m/minute. Prior to the exercise session, and at 1 h and 24 h after the exercise bout, blood samples were taken by tail vein, using 22-gauge needles and heparinised capillary tubes. We have previously shown at these post-exercise times that plasma CK and muscle protein thiol oxidation are increased [28,39]. Blood was centrifuged and plasma stored at −80 °C until biochemical analysis. Just before the time of tail vein sampling, the grip strength of mice was measured using a Chatillon Digital Force Gauge (DFE-002). Mice were placed on the front of the triangle bar (attached to a force transducer) and pulled gently until released. Each mouse underwent 5 consecutive grip-strength trials; the grip strength value for each mouse was recorded as the average of the three trials with the highest force. Average grip strength was normalised to body weight. Mice were sampled 24 h after the exercise protocol.

At termination of the experiment, while mice were under terminal anaesthesia (2% *v*/*v* Attane isoflurane, Bomac, Hallam, VIC, Australia), whole blood was collected via cardiac puncture, immediately centrifuged, plasma removed, and stored at −80 °C until biochemical analysis. Mice were then killed by cervical dislocation and quadriceps muscles were dissected and either frozen in pre-cooled isopentane for histological analysis or snap frozen in liquid nitrogen for biochemical analysis.

### 2.4. Histology

Frozen muscles were cut in transverse sections (8 μm) through the mid-region on a Leica CM3050S cryostat (Leica, Wetzlar, Germany) and stained with Haematoxylin and Eosin (H&E). For morphological analysis, non-overlapping tiled images of transverse muscle sections were acquired with a Nikon Eclipse Ti microscope equipped with a CoolSNAP-HQ2 camera, using Nikon NIS-Elements software (version 4.0, Melville, NY, USA).

Muscle morphology was drawn manually by the researcher using ImageJ software (version 1.53, National Institutes of Health, Bethesda, MD, USA). The area occupied by necrotic myofibres (myofibres with fragmented sarcoplasm and/or areas of inflammatory cells) was measured as a percentage (area) of the whole muscle section. All section analyses were performed ‘blind’.

Serial sections (as per above) were also stained for albumin by immunohistochemistry. Briefly, sections were air-dried and incubated in 2% paraformaldehyde in phosphate buffered saline (PBS), pH 7.2. After washing with tris-buffered saline with Tween-20 (TBST), samples were incubated with 3% hydrogen peroxide. Sections were washed again and incubated overnight at 4 °C with antibodies to albumin (A0433, Sigma-Aldrich) diluted 1:20,000 in TBST. After washing, sections were incubated with horseradish peroxidase conjugated goat anti-rabbit secondary antibodies (Thermo Fisher Scientific, Waltham, MA, USA) diluted 1:1000 in 5% skim milk in TBST for one hour at room temperature. Samples were washed and incubated with 3,3′-Diaminobenzidine (DAB) solution for 15 min. Sections were finally washed with tap water, counter-stained with haematoxylin, dehydrated, cleared, and mounted for microscopy. Digital images were acquired as per above.

### 2.5. Plasma CK

Plasma CK activity reflects the leak of CK from myofibres into the blood and is a classic systemic measure of damage and necrosis of dystrophic muscles [40]. CK levels were measured using a CK-NAC kit (CK110, Randox Laboratories, Crumlin, UK) and analysed kinetically using a BioTek Powerwave XS Spectrophotometer using the Biotek software (Gen 5, Agilent, Santa Clara, CA, USA).

### 2.6. Muscle Inflammation

Myeloperoxidase (MPO) is an enzyme secreted by neutrophils (inflammatory cells that appear very rapidly after tissue damage) and MPO activity is a useful biomarker of neutrophils in tissues [41,42]. The enzyme MPO catalyses the production of hypochlorous acid (HOCl) from hydrogen peroxide and chloride [43] and HOCl acid reacts with 2-[6-(4-aminophenoxy)-3-oxo-3H-xanthen-9-yl]benzoic acid (APF) to form the highly fluorescent compound fluorescein, that is measured in this method, as previously described [29]. Briefly, frozen muscle was crushed under liquid nitrogen and homogenised in 0.5% hexadecyltrimethylammonium bromide in PBS. Samples were centrifuged and supernatants were diluted in PBS. Human MPO was used as the standard for the assay (Cayman Chemical, Ann Arbor, MI, USA). Aliquots of each experimental sample or MPO standard were pipetted into a 384-well plate, before APF working solution (20 µM APF and 20 µM hydrogen peroxide in PBS) was added. The plate was incubated at room temperature (protected from light) for 30 min, with the fluorescence being measured every minute using excitation at 485 nm and emission at 515–530 nm. The rate of change of fluorescence for each sample was compared to that of the standards and results were expressed per mg of protein, quantified using the DC protein assay (Bio-Rad, Hercules, CA, USA).

### 2.7. Muscle Total Protein Thiol Oxidation

Total muscle protein thiol oxidation was measured using the 2-tag technique as described previously [29]. In brief, frozen muscle was crushed under liquid nitrogen before protein was extracted with 20% trichloroacetic acid (TCA)/acetone. Protein was solubilised in SDS buffer and protein thiols were labelled with the fluorescent dye BODIPY FL-N-(2-aminoethyl) maleimide (FLM, Invitrogen, Waltham, MA, USA). Following the removal of the unbound dye using cysteine, protein was re-solubilised in SDS buffer and oxidised thiols were reduced with tris(2-carboxyethyl)phosphine (TCEP) before the subsequent unlabelled reduced thiols were labelled with a second fluorescent dye, Texas Red C2-maleimide (Texas red, Invitrogen). The sample was washed in 100% TCA, followed by acetone, and resuspended in SDS buffer. Samples were read using a fluorescent plate reader (Fluostar Optima, Offenburg, Germany) with wavelengths set at excitation 485 nm and emission 520 nm for FLM and excitation 595 nm and emission 610 nm for Texas red. A standard curve for each dye was generated using ovalbumin and the results were expressed per mg of protein, quantified using the DC protein assay (Bio-Rad).

### 2.8. Muscle Albumin Thiol Oxidation Method Development

**Figure 1:**
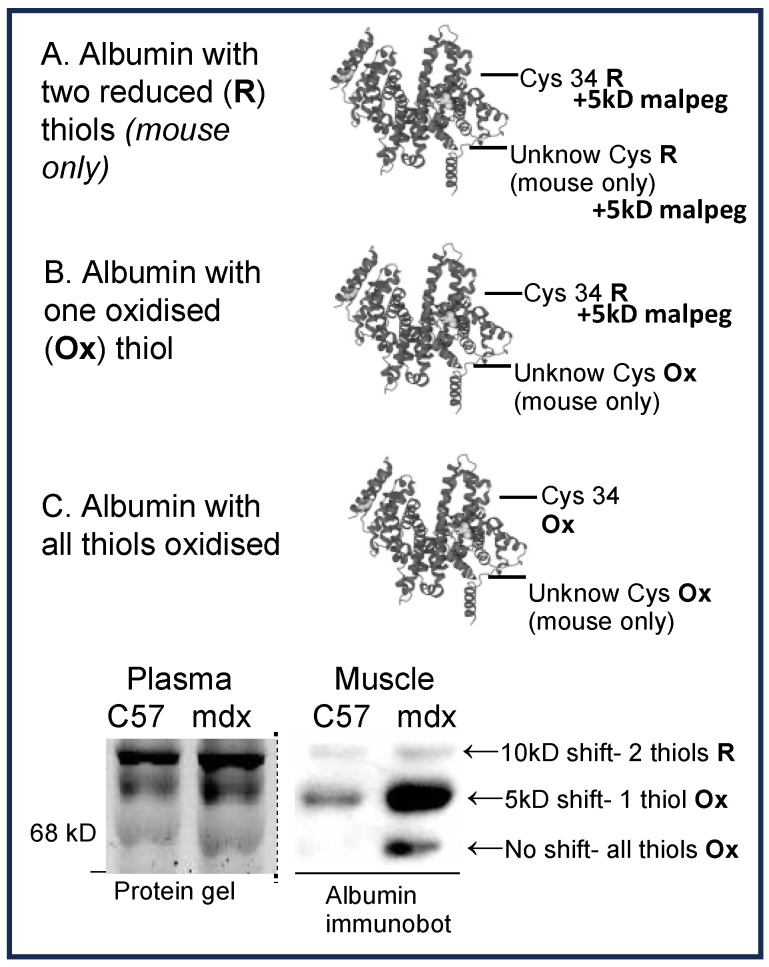
Diagram for immunoblot method to quantify albumin thiol oxidation in plasma and muscle. Plasma and muscle tissue extracts are treated with malpeg that binds to reduced albumin, causing a molecular weight shift that is detectable via immunoblot using antibodies for albumin. Since mouse albumin has two cysteine residues susceptible to redox modifications, albumin can be detected in three states, represented as three distinct bands on the immunoblot membrane. (**A**) represents albumin with two thiols in the reduced (R) state (with two malpeg molecules bound) and a 10 kD shift in band observed. (**B**) represents albumin with only one reduced thiol, and therefore one oxidised (Ox) thiol. This results in only one malpeg molecule bound and a 5 kD shift observed. (**C**) represents albumin with both thiols being oxidised (with no malpeg bound) and no shift is observed. In the Results text, the extent of thiol oxidation (Ox albumin) is referred to as ‘the sum of fully and partially oxidised albumin’ (**B** + **C**) and ‘fully oxidised albumin’ (**C**). Note that in other species, only one cysteine residue (Cys34) is susceptible to redox modifications, and therefore only one shift is observed when Cys34 is in the reduced state. Also of note, in mice, we do not know which thiol group (Cys34 or the other) is more susceptible to oxidation, and therefore which one is highly oxidised in muscle.

It is important to note that while all previous research has indicated that albumin has a total of 35 cysteine residues, with 34 forming 17 intramolecular disulfide bridges with the remaining residue (Cys34) being free and redox-active [7], we have consistently detected two thiols in mouse albumin susceptible to oxidation (confirmed by UniProt sequence). We have not observed this in any other species. We therefore adapted our method to measure oxidised albumin specifically in mouse plasma and modified it further to be used in muscle tissue. These methods are detailed below and illustrated in Figure 1. Malpeg undergoes PEGylation reactions with thiol groups on cysteine side chains and has a large molecular weight (5 kD is used in our method), and therefore it can cause a molecular weight shift that is observable on SDS-PAGE gels and immunoblot. This allows for the separation of oxidised and reduced albumin (since oxidised cysteine cannot bind to malpeg). We were therefore able to establish the oxidation state of albumin in both *mdx* plasma as well as muscle. As illustrated in Figure 1, most albumin in both WT and *mdx* muscle has one thiol oxidised; therefore, we have chosen to present for skeletal muscle just the data for the percentage of albumin with two cysteine side chains being oxidised (expressed as fully oxidised albumin).

In contrast, for plasma albumin thiol, a significant amount of albumin has two reduced thiols in both WT and *mdx* mice. Therefore, for plasma, we present the sum of the percentage of albumin with both one and two thiols oxidised (which we have termed ‘sum of fully and partially oxidised albumin’) and two thiols oxidised (fully oxidised).

### 2.9. Muscle Albumin Thiol Oxidation Method

Analysis of muscle albumin thiol oxidation (non-mercaptalbumin-1 and 2) was performed using our method for analysis of plasma samples with some modifications [28]. Although plasma albumin thiol oxidation could be measured using electrophoresis (see below), the method was not sufficiently sensitive for muscle and therefore an immunoblot with increased sensitivity was used. Frozen muscle samples were crushed under liquid nitrogen, and aliquots of approximately 10 mg were homogenised in PBS containing 1 mM methoxypolyethylene glycol maleimide (malpeg, 5000 g/mol, JenKem Technology) malpeg (previously diluted in 40 mM imidazole, pH 7.4). In order to remove excess malpeg (which interferes with electrophoresis), protein was extracted using a chloroform-methanol protein precipitation method. Extracted protein was resuspended in loading buffer containing 94 mM Tris pH 7, 2% SDS, 0.015% bromophenol blue, 15% glycerol, and 350 mM dithiothreitol (DTT), and it was heated for 15 min at 80 °C. Samples were resolved in 12% acrylamide and 1% SDS gels containing 1% (*v*/*v*) of 2,2,2-trichloroethanol for fluorescent stain-free imaging [44]. Gels were imaged using the Stain-Free imaging program on the ChemiDoc MP Imaging System (Bio-Rad), and loading was checked by measuring albumin signal. Proteins were transferred to nitrocellulose membranes using the Trans Turbo Blot System (Bio-Rad). Immunoblotting was performed with antibodies to albumin (A0433, Sigma-Aldrich) dissolved 1:60,000 in tris-buffered saline (TBST). Horseradish peroxidase conjugated goat anti-rabbit secondary antibodies (Thermo Fisher Scientific) were diluted 1:10,000 in 5% skim milk in TBST. The ChemiDoc MP Imaging System (Bio-Rad) was used to capture chemiluminescence signals. ImageJ software (version 1.53) was used to quantify the resultant images [45]. Total albumin content was standardised to total protein (as measured by stain-free imaging).

Sample analysis for irreversible albumin oxidation (non-mercaptalbumin-2) was also performed by reducing malpeg labelled as per above with L-cysteine hydrochloride monohydrate, which was added to a final concentration of 3 mM, followed by incubation at room temperature for 60 min. Malpeg was added to a final concentration of 3.5 mM, followed by incubation at room temperature for 15 min. Excess malpeg was removed and immunoblotting was performed as above. We observed that in both WT and *mdx* muscle, the majority of albumin had one cysteine residue undergoing irreversible oxidation, while neither WT nor *mdx* had both cysteine residues undergoing irreversible oxidation. It was therefore determined that in muscle, the measurement of irreversible albumin cysteine oxidation was not a useful measure of dystropathology.

### 2.10. Plasma Albumin Thiol Oxidation

Plasma albumin thiol oxidation was measured using our established method [28], adapted for capillary electrophoresis (CE). In brief, plasma samples were incubated with 30 mM malpeg for 15 min. Malpeg conjugated plasma was aliquoted into two: (1) aliquot-1 was diluted with CE-buffer (0.01% DMSO, 45 mM phosphate pH 8.0 with 2.5% *w*/*v* SDS) and (2) aliquot-2 was reduced for irreversible albumin oxidation analysis. The reduction of reversibly oxidised thiols from aliquot-2 was performed by incubation with 7.5 mM cysteine for 30 min. Malpeg was added to a final concentration of 15 mM, followed by incubation for 15 min. Reduced samples were diluted with CE-buffer.

Capillary electrophoresis was performed using an Agilent 7100 CE system. The capillary used was a 50-micron bare-fused silica capillary (Polymicro Technologies, Tucson, AZ, USA) with an effective length of 32.5 cm. The background electrolyte was composed of 45 mM phosphate pH 8.0 with 2.5% SDS. Diluted samples were hydrodynamically (10 mbar/s for 10 s) injected, with electrophoretic separation performed by applying +15 kV for 15 min. Detection was by absorbance at a wavelength of 214 nm. Area under the curve analysis was performed using Agilent Chemstation software (version B.04.03).

### 2.11. Statistics

Significant differences between groups were determined using GraphPad Prism software (Version 9.4.1). Data were analysed using two-way ANOVA tests with post hoc testing. All data are presented as mean ± standard error of the mean (SEM). Significance was set at *p* < 0.05. Pearson’s correlation was used to assess the relationship between plasma and muscle albumin thiol oxidation and dystropathology measures.

## 3. Results

### 3.1. Characterisation of Dystropathology

**Figure 2:**
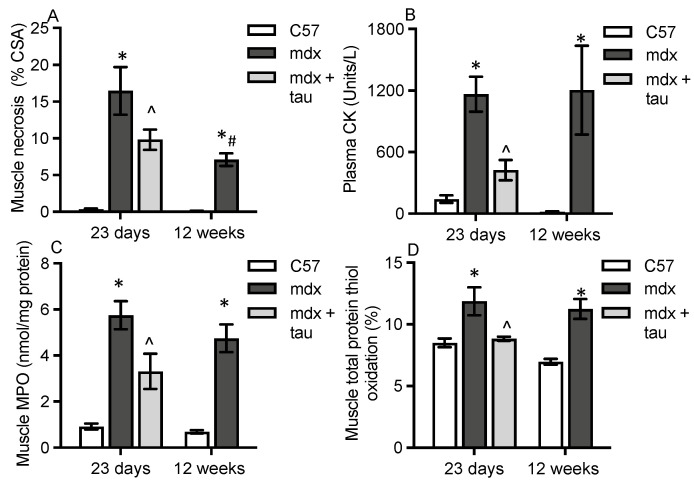
Measures of dystropathology. Myofibre necrosis (**A**), plasma CK (**B**), muscle inflammation, myeloperoxidase (MPO, **C**), and total protein thiol oxidation (**D**) in young 23-day-old and adult 12-week-old WT and untreated *mdx* mice, plus for taurine-treated young *mdx* mice sampled at 23 days. CSA= cross-sectional area. * = significantly (*p* < 0.05) different to WT of same age. ^ = significantly (*p* < 0.05) different to untreated *mdx* (effect of taurine treatment). # = significantly (*p* < 0.05) different to same strain at 23 days. Bars represent mean ± SEM and n = 7–8 mice per group.

To examine the effect of changes in dystropathology on albumin oxidation in the muscle and blood of *mdx* mice, we tested young mice at the time of peak active myonecrosis (23 days old) and young adults where there is less active myonecrosis (12 weeks). Myofibre necrosis was 16-fold higher in quadriceps of 23-day-old *mdx* muscle versus WT and 20-fold higher in 12-week-old *mdx* muscle versus WT (Figure 2A). Myofibre necrosis was 1.6-fold higher in quadriceps of 23-day-old compared with 12-week-old *mdx* mice (Figure 2A).

Another classic marker of dystropathology, plasma CK content measuring myofibre membrane leakiness, was 12-fold higher in 23-day-old mdx versus WT mice and 24-fold higher in 12-week-old *mdx* versus WT mice (Figure 2B), with no significant difference between the young and adult *mdx* plasma CK levels (Figure 2B). The characterisation of dystropathology by increased inflammation and oxidative stress was evident in *mdx* quadriceps muscles at both ages, compared with WT mice as shown by increased MPO activity, a marker of neutrophil presence, and protein thiol oxidation, a marker of oxidative stress. The MPO activity in quadriceps muscles was 5.5–6-fold higher in *mdx* compared with WT mice (Figure 2C), with no significant impact of age. Similarly, total protein thiol oxidation in quadriceps was 1.4–1.5-fold higher in *mdx* compared with WT mice, with no impact of age (Figure 2D).

Short-term treatment of juvenile *mdx* mice with taurine (from 15–23 days), compared with untreated control *mdx* mice, decreased myonecrosis by 38% (Figure 2A), plasma CK content by 60% (Figure 2B), muscle MPO activity by 45%, and muscle protein thiol oxidation by 25% (Figure 2C,D). This therapeutic intervention further demonstrates a strong association between these four different measurements of dystropathology in muscle and blood. This combined data in young and adult *mdx* mice form the background for the following in-depth analysis of protein thiol oxidation of albumin in dystrophic plasma and muscle. Taken together, these data show that 23-day-old and 12-week-old *mdx* mice were suitable models to examine the effects of dystropathology on albumin oxidation.

### 3.2. Albumin Thiol Oxidation in mdx Plasma

**Figure 3:**
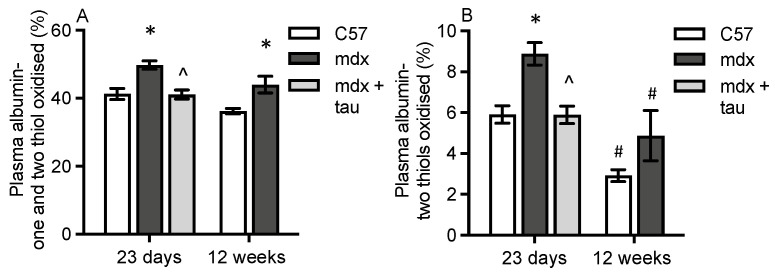
Levels of oxidised plasma albumin. Both the sum of fully and partially oxidised plasma albumin (**A**) and fully oxidised plasma albumin (**B**) in 23-day-old and 12-week-old WT and untreated *mdx* mice, and taurine-treated *mdx* mice aged 23 days. * = significantly (*p* < 0.05) different to WT of same age. ^ = significantly (*p* < 0.05) different to untreated *mdx* (effect of taurine treatment). # = significantly (*p* < 0.05) different to same strain at 23 days. Bars represent mean ± SEM and n = 7–8 mice per group.

Two forms of oxidised albumin were measured as summarised in Figure 1: partially oxidised albumin (one thiol oxidised) and fully oxidised albumin (two thiols oxidised). In plasma, the sum of partially and fully oxidised albumin was 1.2-fold higher in both young and adult *mdx* mice, compared with WT, with no impact of age in either strain (Figure 3A).

Fully oxidised albumin was 1.5-fold higher in the plasma of young (23 day) *mdx* mice compared with WT (Figure 3B). However, for adult (12 week) mice, there was no difference between strains. Levels of fully oxidised albumin were 50% lower in both adult strains, compared with young mice of the same strain (Figure 3B). These data indicate that oxidation of plasma albumin was particularly pronounced during periods of active myonecrosis (23-day-old young *mdx* mice). Taurine treatment of juvenile *mdx* mice for 7 days significantly reduced the sum of partially and fully oxidised albumin by 15% and 33%, respectively, in 23-day-old mice (Figure 3A,B).

### 3.3. Levels of Albumin in Dystrophic and Normal Muscles

**Figure 4:**
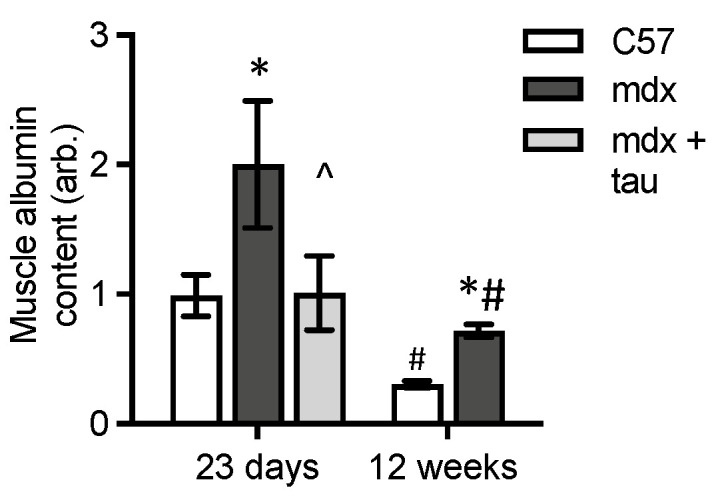
Levels of albumin protein in muscles. Albumin content in quadriceps muscles of 23-day-old and 12-week-old WT and untreated *mdx* mice and taurine-treated *mdx* mice aged 23 days. * = significantly (*p* < 0.05) different to WT of same age. ^ = significantly (*p* < 0.05) different to untreated *mdx* (effect of taurine treatment). # = significantly (*p* < 0.05) different to same strain at 23 days. Bars represent mean ± SEM and n = 7–8 mice per group.

Dystrophy causes extensive tissue disturbances involving the vasculature, myonecrosis and inflammation which have the potential to affect albumin levels within the muscle, and exposure to oxidants. We therefore measured the levels of albumin in skeletal muscle. In mdx muscles, albumin content was higher (about 2-fold) at both ages, compared with WT (Figure 4).

Albumin content was approximately 3-fold higher in quadriceps of 23-day-old compared with 12-week-old WT and *mdx* mice (Figure 4). Taurine treatment prevented the increase in albumin content seen at 23 days in untreated *mdx* muscle (Figure 4), reflecting the prevention of myonecrosis, with albumin levels being similar to normal WT muscle.

**Figure 5:**
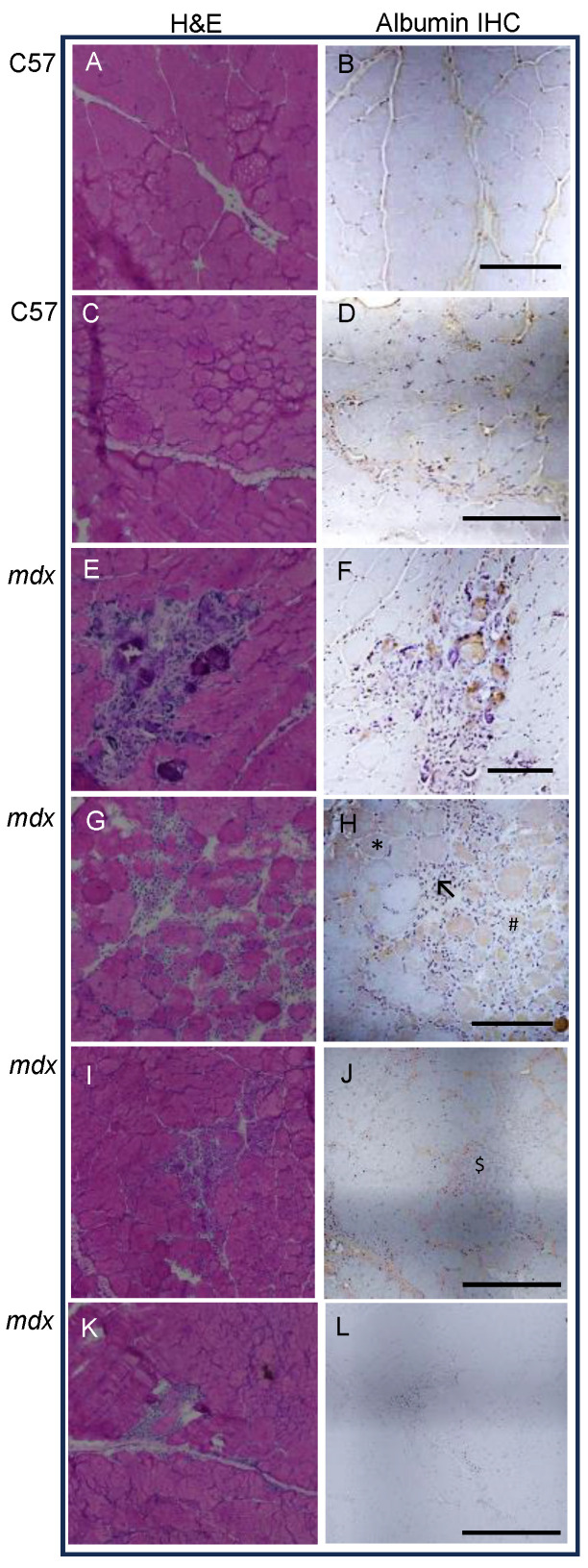
Location of albumin in muscles, visualised by immunostaining. Serial frozen sections of 23-day-old WT (**A**–**D**) and *mdx* (**E**–**J**) quadriceps muscle were stained with H&E (**A**,**C**,**E**,**G**,**I**,**K**) and with antibodies to albumin (**B**,**D**,**F**,**H**,**J**). Section (**L**) is a negative control (no primary antibody). In *mdx* muscle, albumin staining is in the areas of immune cell recruitment (arrow), and within necrotic myofibres, including hypercontracted (asterisk), degenerated (hash), and newly formed (dollar). Images were taken at 10× magnification and scalebar line = 200 µm.

Since albumin content was about 2-fold higher in (untreated) *mdx* quadriceps muscle at both ages (compared with WT mice), the location of albumin within the muscles was investigated by immunostaining using albumin antibodies. Albumin in WT muscles was present in the interstitium outside the myofibres (as expected) since albumin does not enter intact myofibres (Figure 5B,D).

In *mdx* muscle as well as the interstitium, there was increased staining of albumin in areas of myonecrosis, also in the interstitium where immune cells were present, and there was inflammation within myofibres that were undergoing necrosis (Figure 5F,H,J). This albumin staining is observed in hypercontracted myofibres (indicating an early stage of necrosis), myofibres that are fully degenerated, and some newly regenerating myofibres (Figure 5F,H,J).

### 3.4. Levels of Albumin Thiol Oxidation in mdx Muscle

**Figure 6:**
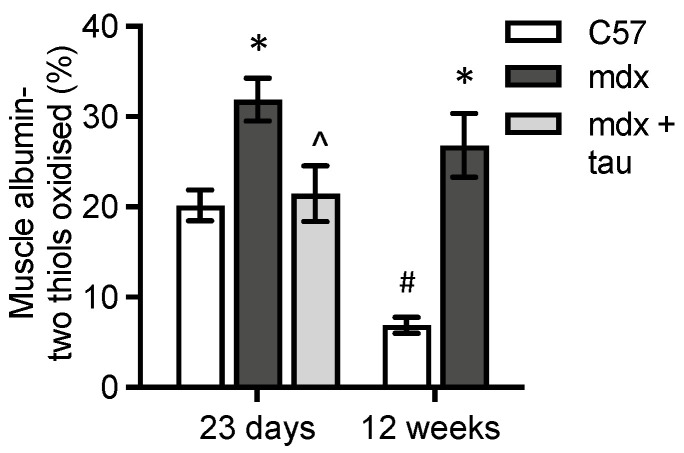
Fully oxidised albumin in quadriceps muscle. Albumin in 23-day-old and 12-week-old WT and untreated *mdx* mice and taurine-treated *mdx* mice aged 23 days. * = significantly (*p* < 0.05) different to WT of same age. ^ = significantly (*p* < 0.05) different to untreated *mdx* (effect of taurine treatment). # = significantly (*p* < 0.05) different to same strain at 23 days. Bars represent mean ± SEM and n = 7–8 mice per group.

We examined whether albumin in muscle was oxidised given that dystrophic muscle is characterised by increased oxidative stress (Figure 2D). Fully oxidised albumin was 1.6-fold higher in 23-day-old *mdx* muscle versus WT and 4-fold higher in 12-week-old *mdx* muscle versus WT (Figure 6).

Fully oxidised albumin was 65% lower in 12-week-old WT muscle compared to 23-day-old WT muscle, but there was no difference in fully oxidised albumin between 23-day-old and 12-week-old adult *mdx* muscle (Figure 6). Taurine treatment decreased fully oxidised albumin in 23-day-old *mdx* muscle by 40% (Figure 6).

We compared the extent of albumin oxidation in plasma and muscle tissue by comparing the amount of fully oxidised albumin in plasma and muscle shown in Figure 1B and Figure 6. In 23-day-old and 12-week-old WT mice, fully oxidised albumin was 3.3-fold and 2.5-fold higher in muscle tissue, relative to plasma. This difference was also observed with *mdx* mice, where fully oxidised albumin was 3.4-fold, 3.5-fold, and 5-fold higher in 23-day-old mice, 23-day-old mice treated with taurine, and 12-week-old *mdx* mice. Together, these data indicate that in mice, fully oxidised albumin is consistently higher in muscle than plasma.

To assess whether changes in muscle albumin thiol oxidation are associated with increased necrosis and inflammation, we compared thiol oxidation with other measures of damage and inflammation (in both strains). All indices (muscle albumin, total muscle protein thiol oxidation, myofibre necrosis, muscle inflammation, and plasma CK content) correlated significantly with plasma and muscle albumin thiol oxidation; with plasma and muscle albumin thiol oxidation also correlated with each other (Table 1).

### 3.5. Tracking Plasma Albumin Thiol Oxidation

We have previously proposed that plasma albumin thiol oxidation could be used to track acute changes in dystropathology (such as after the initiation of a drug treatment) [46]. Therefore, we tested changes in albumin thiol oxidation (and other measures of dystropathology including plasma CK and muscle strength) in individual 12-week-old *mdx* mice that underwent a treadmill exercise session, known to exacerbate dystropathology. Tail vein blood samples were taken before, 1 h after, and 24 h after a single 30 min treadmill exercise session. These time points were chosen as we have previously shown that plasma CK and muscle protein thiol oxidation are increased [28,39]. CK and grip strength were used as established biomarkers of muscle pathology to track the effects of exercise.

**Figure 7:**
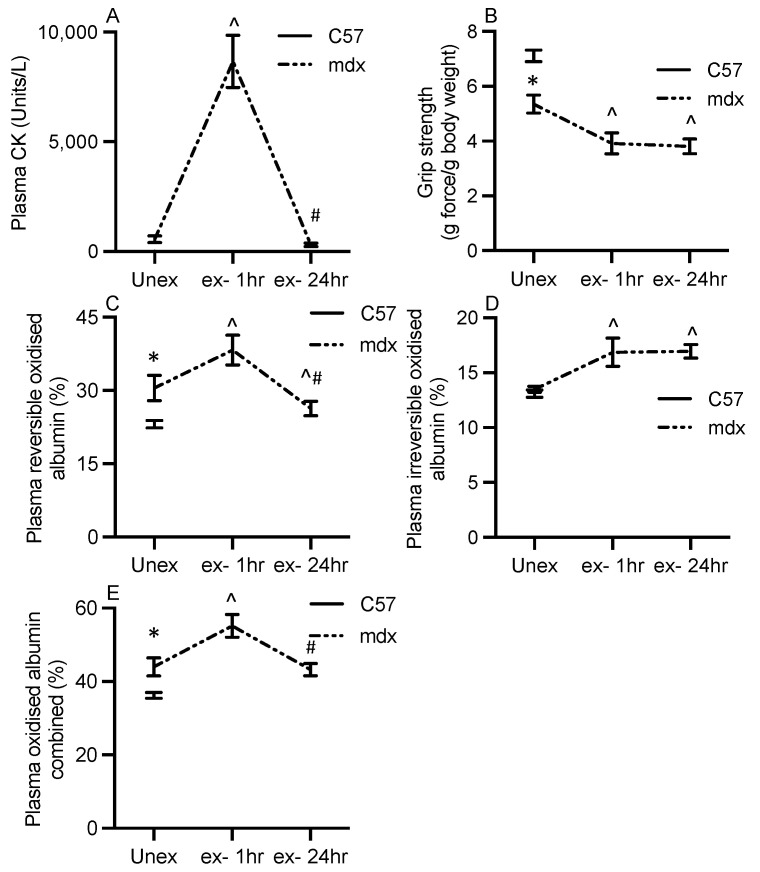
Plasma albumin thiol oxidation and measures of dystropathology in serial samples of treadmill-exercised *mdx* mice. Graphs show data from unexercised WT mice, and *mdx* mice pre-exercise and with sampling at 1 h and 24 h after a single 30 min treadmill exercise for plasma CK (**A**), grip strength (**B**), plasma reversibly oxidised albumin (**C**), plasma irreversibly oxidised albumin (**D**), and the combination of reversibly and irreversibly oxidised albumin (**E**). * = significantly (*p* < 0.05) different to WT of same age. ^ = significantly (*p* < 0.05) different to pre-exercised *mdx* values. # = significantly (*p* < 0.05) different to 1 h post-exercise *mdx* values. Bars represent mean ± SEM and n = 6–8 mice per group.

CK content was 10-fold higher in *mdx* versus WT plasma (pre-exercised, Figure 7A).

One hour post-exercise, *mdx* plasma CK content was 14-fold higher than baseline (pre-exercised, Figure 7A). At 24 h post-exercise, CK content in *mdx* plasma had returned to baseline (Figure 7A). Grip strength was 20% lower in *mdx* versus WT (Figure 7B). At both 1 h and 24 h post-exercise, grip strength was 25% lower than baseline (pre-exercised, Figure 7B).

Since differences in irreversible oxidation levels of plasma albumin were observed when comparing *mdx* and WT mice, data are presented as reversible, irreversible, and both combined. Reversibly oxidised plasma albumin was 1.2-fold higher in *mdx* versus WT plasma (Figure 7C). One hour post-exercise, reversibly oxidised albumin in *mdx* plasma was 1.3-fold higher than baseline (pre-exercised, Figure 7C); and by 24 h post-exercise, this had returned to baseline (Figure 7C). Irreversibly oxidised albumin in plasma was the same in (unexercised) *mdx* and WT mice (Figure 7D). At 1 h post-exercise, irreversibly oxidised albumin was 1.2-fold higher than baseline (pre-exercised, Figure 7D) and remained high at 24 h (i.e., did not return to baseline levels in contrast with reversibly oxidised plasma albumin). When combining the reversible and irreversible data, combined oxidised albumin was 1.2-fold higher in (unexercised) *mdx* plasma versus WT (Figure 7E). At 1 h post-exercise, combined *mdx* oxidised albumin was 1.3-fold higher than baseline (pre-exercised, Figure 7E) and at 24 h post-exercise, it had returned to baseline (Figure 7E).

## 4. Discussion

The new data presented here reinforce the relationship between plasma and muscle albumin thiol oxidation, as well as measures of ongoing dystropathology in *mdx* mice. The key results are the following: (i) a new method is described to measure albumin thiol oxidation in tissues utilising immunoblot. The use of this new method shows (ii) that levels of muscle albumin thiol oxidation in dystrophic *mdx* plasma and muscle are significantly higher than normal control at two ages, 23 days and 12 weeks, and these data correlate with measures of dystropathology, including global protein thiol oxidation, myofibre necrosis, muscle inflammation, and plasma CK release. We also demonstrate (iii) that plasma albumin thiol oxidation measured serially in *mdx* mice can track acute changes in dystropathology induced experimentally by an acute bout of exercise in adult mice. The significance of these combined new observations is discussed below.

Oxidative stress has been implicated in the pathology of DMD [47], so the accurate measurement of oxidation in dystrophic tissue can provide insight into molecular mechanisms causing pathology. We have previously developed a method (2-tag) to measure the global protein thiol oxidation in tissue, using two different maleimide tags that measure reduced and oxidised protein thiols [48]. This method has been used to successfully measure oxidative stress in skeletal muscle in the *mdx* mouse model of DMD [29]. We have found this to be an accurate and sensitive measure of oxidative stress; however, the method is challenging and requires training. We have also previously developed a sensitive oxidative stress method for plasma albumin thiol oxidation, which can be performed with immunoblot [28]. We therefore adapted this method to measure albumin thiol oxidation in tissue utilising immunoblot, making it a relatively easy to perform and a readily accessible method for use as a sensitive marker of oxidative stress in muscle.

Of note is that previous research suggests that albumin has a total of 35 cysteine residues with 34 forming 17 intramolecular disulfide bridges, and the remaining residue (Cys34) is free and redox-active [7]. However, we consistently detected two thiols in mouse albumin susceptible to oxidation but not in any other species we have investigated, including humans, rats, dogs, cows, horses, and sheep. Interestingly, and unlike in plasma, we found substantial irreversible thiol oxidation (non-mercaptalbumin-2) on one cysteine residue only in muscle, even in normal WT mice. It is not known if this irreversible oxidation is occurring on this additional cysteine or on Cys34. Since we have not been able to find any other studies that have also observed this additional thiol group on mouse albumin, nor any studies that have observed this extensive irreversible oxidation of albumin in muscle or other tissue, we are not able to further interpret the biological significance of these observations. Additional mass spectrometry analysis of mouse albumin, from both plasma and within tissue, is required to better understand the type of irreversible oxidation. The analysis of the extent of irreversible albumin thiol oxidation in muscle from other species (where this additional Cys residue is not observed) may also give some indication of the significance of this new result in mice.

An interesting observation was that the age of mice (*mdx* and normal WT) had an impact on levels of both plasma and muscle albumin thiol oxidation, with levels of fully oxidised albumin initially high in juveniles at 23 days and then decreased by 12 weeks (in adults). We have previously observed this in both *mdx* and WT plasma, with albumin thiol oxidation decreasing after 23 days and increasing again by 18 months [28]. Increased oxidation of albumin in aging has been reported previously [49], but the high levels of albumin oxidation in juvenile mice appear to be a novel observation as we were not able to locate any literature reporting this occurrence. Further work is required to establish the biological significance of the higher level of albumin oxidation during early post-natal growth and whether this is a consequence of increased generation of oxidants or decreased antioxidant activity.

While albumin is abundant in plasma, most body albumin is in the extravascular compartment of tissues such as muscle, skin, and adipose tissue [1,2]. Studies show the substantial transcapillary transit of albumin with return to the plasma compartment via the lymphatic system [2,50]. With exercise, there is a substantial increase in albumin content, and albumin transit, in muscle [51]. We therefore hypothesised that changes in the extent of plasma albumin thiol oxidation are a consequence of changes in the oxidative state of albumin in tissues, specifically skeletal muscles that comprise about 40% of body mass. A novel observation was that even in normal WT muscles, the amount of oxidised albumin is considerably (approximately 4-fold) higher than for plasma, suggesting that the movement of albumin through tissue is a significant source of oxidation. This may be particularly true for muscle, which produces significant oxidants due to its contractile activity and high oxygen consumption [52].

We found in *mdx* muscle that albumin content was increased (relative to normal muscle), and this is consistent with reports that inflammation increases albumin content, and albumin transit, in tissues [53]. In *mdx* muscles, albumin was high in the interstitium (especially around areas of inflammatory cell invasion), and also within myofibres. Many studies show that albumin (bound to Evans blue dye) accumulates in dystrophic myofibres after exercise-induced damage [54,55]. Since it is visually confirmed that protein oxidation is mainly colocalised to areas of myofibre necrosis and associated immune cell infiltration in *mdx* muscles [26], the high levels of albumin localised in these areas of dystrophic muscle damage are likely to become oxidised within this cellular environment [26]. The present study also demonstrated that the oxidation of albumin is prevented by taurine administration, an intervention widely reported to reduce dystropathology, including decreased myonecrosis and inflammation [29,30,31,32,33,34,35,36,37]. Interestingly, the present study also showed that taurine decreased the amount of albumin entering the muscle, supporting the hypothesis that the increased albumin thiol oxidation in *mdx* muscle (and therefore plasma) is a consequence of increased albumin migrating through the muscle tissue.

To further examine whether plasma albumin thiol oxidation could be used as a biomarker to track acute changes in dystropathology in individual *mdx* mice, we subjected adult (12 week) *mdx* mice to a single treadmill exercise session, which is known to increase myonecrosis and plasma CK release and decrease grip strength [39]. Our findings revealed two patterns of plasma albumin oxidation, with both reversible and irreversible albumin thiol oxidation rapidly elevated at 1 h after exercise, with reversible, but not irreversible, plasma albumin thiol oxidation returning to baseline by 24 h after exercise. This difference may reflect irreversible oxidation of albumin being a permanent modification to the protein, whereas reversible oxidation can be reversed by thiol/disulfide exchange [11,56,57]. Thus, the exercise caused acute changes in pathology associated with the very rapid increase in oxidative stress within the muscles, that oxidised the local albumin in the muscle tissues, that then returned to the circulating plasma, which caused the change in albumin reversible oxidation. This concept is supported by the pattern of plasma CK release, which was similar (with a rapid increase within one hour) to total plasma albumin thiol oxidation. As changes in CK reflect increased permeability of the muscle plasma membrane (sarcolemma) caused by dystropathology, the changes in reversible thiol oxidation likely also reflect acute changes in dystropathology. In contrast, the sustained decrease in grip strength (loss of function) indicates ongoing damage which may be related to a sustained increase in oxidants having direct adverse effects on myosin and other contractile proteins [58] and causing the additional irreversible oxidation of albumin.

## 5. Conclusions

We show that albumin thiol oxidation is elevated in plasma of *mdx* mice, and this reflects increased albumin oxidation in *mdx* muscles, plus plasma albumin thiol oxidation is acutely responsive to changes in dystropathology. These combined observations strongly support the measurement of changes in plasma albumin oxidation as a promising biomarker to track acute changes in dystropathology. There is a need for molecular biomarkers to track the severity of dystropathology in animal models and DMD, particularly for blood biomarkers to help rapidly evaluate the potential efficacy of clinical trials for DMD.

## Figures and Tables

**Table 1:** Correlation between plasma and muscle for fully oxidised albumin (see definition in Figure 1) and measures of dystropathology. Data shown for 23-day-old and 12-week-old *mdx* and WT control mice. N = 37. *r* = Pearson correlation coefficient. * represents significant correlation of *p* < 0.05.

Indices 1	Indices 2	*r*	*p*
Oxidised plasma albumin	Oxidised muscle albumin	0.8	<0.0001 *
Oxidised plasma albumin	Muscle albumin	0.5	<0.02 *
Oxidised plasma albumin	Total muscle protein thiol oxidation	0.6	<0.0003 *
Oxidised plasma albumin	Muscle necrosis	0.6	<0.0003 *
Oxidised plasma albumin	Muscle inflammation	0.6	<0.0003 *
Oxidised plasma albumin	Plasma Ck	0.7	<0.0001 *
Oxidised muscle albumin	Muscle albumin	0.6	<0.001 *
Oxidised muscle albumin	Total muscle protein thiol oxidation	0.6	<0.003 *
Oxidised muscle albumin	Muscle necrosis	0.6	<0.006 *
Oxidised muscle albumin	Muscle inflammation	0.6	<0.0001 *
Oxidised muscle albumin	Plasma Ck	0.7	<0.0001 *

## Data Availability

The original contributions presented in the study are included in the article, further inquiries can be directed to the corresponding author/s.
